# Cannabis use and its association with psychopathological symptoms in a Swiss adult population: a cross-sectional analysis

**DOI:** 10.3389/fpubh.2024.1356988

**Published:** 2024-05-22

**Authors:** Christoph Felix Mosandl, Lavinia Baltes-Flückiger, Jens Kronschnabel, Maximilian Meyer, Adrian Guessoum, Oliver Herrmann, Marc Vogel, Marc Walter, Eva-Maria Pichler

**Affiliations:** ^1^Clinic of Psychiatry and Psychotherapy, Psychiatric Services Aargau, Windisch, Switzerland; ^2^Department of Addictive Disorders, University Psychiatric Clinics Basel, Basel, Switzerland

**Keywords:** cannabis use, depression, ADHD, cannabis use disorder, cannabis legalization, mental health, cannabis policy

## Abstract

**Background:**

As the most commonly used illicit substance, cannabis is gaining global acceptance through increasing legalization efforts. This shift intensifies the need for research to guide policymakers and healthcare providers in harm reduction and treatment strategies. Nonetheless, the relationship between psychopathological symptoms and cannabis use remains inadequately understood.

**Methods:**

A sample of regular cannabis consumers completed self-reported assessments for depression (Patient Health Questionnaire-9), anxiety (General Anxiety Disorder-7), Attention-Deficit/Hyperactivity Disorder (ADHD; Adult ADHD Self-Report Scale V1.1), and psychosis (Early Recognition Inventory based on IRAOS) as well as previous black-market cannabis use patterns. Cannabis Use Disorder Identification Test Revised (CUDIT-R) was used to identify cannabis use disorder (CUD). To understand psychopathological symptom load related to cannabis consumption as well as cannabis use motives, multiple regression models were performed to identify psychopathological variables predicting cannabis use frequency and quantity. Linear regression and correlation analyses were conducted, adjusting for relevant covariates (age, gender, education, alcohol, other substance use).

**Results:**

Three-hundred-sixty regular cannabis users interested in a study on regulated cannabis access in Basel, Switzerland were examined. In bivariate analysis, cannabis use frequency correlated with depressive (*r*(358) = 0.16, *p* = 0.003) and anxiety symptom load (*r*(358) = 0.11, *p* = 0.034). Cannabis quantity correlated with depressive (*r*(358) = 0.15, *p* = 0.005), ADHD (*r*(358) = 0.14, *p* = 0.008), and psychosis symptom load (*r*(358) = 0.16, *p* = 0.002). However, in the adjusted regression models only depressive and ADHD symptom loads were significantly associated with cannabis use frequency (*p* = 0.006 and *p* = 0.034, respectively) and quantity (*p* = 0.037 and *p* = 0.019, respectively). No significant correlations between cannabis consumption and anxiety or psychosis remained after adjustment.

**Conclusion:**

ADHD and depressive symptoms correlate with increased cannabis use in a cohort of regular users, suggesting potential self-medication in nonclinical populations. With the rising availability of cannabis worldwide, these results highlight the necessity for longitudinal studies to disentangle the complex dynamics between cannabis consumption and mental health symptoms.

## 1 Introduction

Cannabis is the most commonly consumed illicit substance, and its use is growing as attitudes toward legalization shift globally (1). Policymakers and healthcare providers increasingly rely on empirical data to make informed decisions about harm reduction and treatment options. However, the relationship between psychopathological symptoms and cannabis use remains poorly understood. Particularly, little is known about how the quantity of consumed cannabis correlates with psychopathological variables.

In the European Union alone, 22.6 million people, or 8% of the population aged 15 to 64, used cannabis at least once in the past year. This prevalence is even more pronounced among 15- to 24-year-olds, with an annual usage rate of 18.2% (2). Nearly half of treatment-seeking individuals who use cannabis report daily usage. Cannabis use is consistently increasing across Europe, a trend potentially influenced by the rapid pace of legalization efforts (1).

Previous research shows stable associations between cannabis use and mental health disorders (3) in a dose-dependent manner (4). The most robust evidence exists for psychosis, for which heavy cannabis users have a four-fold increased risk compared to non-users (5, 6). Elevated risks have also been demonstrated for depressive (7) and anxiety disorders (8). Additionally, a link between ADHD and cannabis use has been suggested, where problematic use (i.e., associated with health, financial, legal, social issues) was associated with more severe symptoms (9, 10). These specific disorders—psychosis, depression, anxiety, and ADHD—are the focus of the present study, as they represent areas in which cannabis use and mental health intersect the most.

While the direction of the relationship between cannabis use and non-psychotic disorders remains inconclusive, it is known that individuals with mental health disorders are more likely to use or misuse cannabis (11–14).

The psychoactive effects of cannabis are largely explained by tetrahydrocannabinol (THC) concentration. As the amount of cannabis consumed and the THC concentration may vary, complicating the measurement of exposure (15). As of today, only a small number of studies employed quantity as a measure in relationship with psychopathological outcomes (16). Using cannabis more than once a week and high-potency subtypes can lead to cannabis use disorder (CUD) (17, 18).

The present literature mostly focuses on the relationship between cannabis use and acute or chronic mental illnesses. However, a large subgroup of users suffers only from minor mental health impairments and does not meet the diagnostic criteria of a psychiatric disorder (19–21). Additionally, the focus on negative implications may be one-sided, as people suffering from depression, anxiety, ADHD, or psychosis may use cannabis to mitigate symptoms (22–24).

Medical cannabis is mainly prescribed to alleviate symptoms such as anxiety, insomnia, depression, and pain (25, 26). It is equally important to understand the patterns and motivations behind regular non-medical cannabis use despite widely acknowledged risks related to consumption. Regulated cannabis markets may facilitate data collection on the frequency and quantity of consumed cannabis (27), as research on how cannabis quantity impacts mental health is scarce (16). In response to widespread cannabis use and an active black market, Switzerland amended the Narcotics Act (NarcA) in 2021, allowing 10-year pilot trials on non-medical cannabis use in adults. These trials aim to inform future regulatory frameworks and are distinct from traditional medical cannabis use, which remains tightly regulated.

The current study is based on a pilot project providing regulated cannabis access to 360 participants in a major central European city. Making use of the hitherto underutilized measure of consumed cannabis quantity may give important insights, particularly in the context of global increases in consumption and legalization (28).

Given the complexity of findings in existing literature suggesting rather a bidirectional relationship between mental health symptoms and cannabis use, we aim to explore whether among adult cannabis users, the level of symptoms of depression, anxiety, ADHD, and psychosis may be associated with the frequency and quantity of cannabis used in the last 30 days. Our study acknowledges the potential for both directions of influence. The present study aims (a) to provide a sociodemographic description of a population of non-medical cannabis users (confirmed by THC drug test) from a major central European city; (b) to describe the patterns and motives of cannabis use; (c) to investigate the relationship between psychopathological variables and self-reported frequency as well as quantity of cannabis used in the last 30 days.

## 2 Materials and methods

### 2.1 Design

The present cross-sectional study is part of a single-center randomized controlled clinical trial (RCT) called WeedCare (NCT05522205), which aims to investigate the effects of regulated cannabis access on consumption behavior and mental and physical health in comparison to the illicit market. The study has been approved by the local Ethics Committee (Ethikkommission Nordwest- und Zentralschweiz (EKNZ)), as well as by the Swiss Federal Public Health Office, and is conducted in accordance with the Declaration of Helsinki and the ICH-GCP guidelines. Participants were randomly assigned to one of two groups: Group 1 has immediate access to the regulated cannabis sale in pharmacies, whereas Group 2 continues to purchase cannabis on the illicit market. After 6 months all participants also gained access to regulated cannabis in pharmacies for two consecutive years, ensuring consistent quality and THC content for all. Cannabis pricing was subsidized throughout, mirroring illicit market rates to facilitate realistic consumption scenarios. Every 6 months, participants are invited to answer online questionnaires on their cannabis consumption behavior as well as their mental and physical health. The entire study duration is 2.5 years. The detailed study protocol has been published elsewhere (29). Importantly, the present article covers only the baseline data of the study. Results on the regulated cannabis access for recreational use will be presented in future articles.

### 2.2 Eligibility criteria

Individuals eligible for inclusion in the study provided written consent, were 18 years or older, and demonstrated monthly cannabis use over the past 6 months as confirmed by THC in urinalysis. Participants also needed to have sufficient proficiency of the German language as well as internet access to complete the required questionnaires (29). In agreement with local law, only residents of the canton Basel-Stadt were eligible for participation. Exclusion criteria were current psychiatric hospitalization, acute suicidality or psychosis, severe cognitive impairment that could preclude informed consent, and pregnant or breastfeeding women. Eligibility was contingent on exclusion criteria assessed through comprehensive face-to-face screenings with a study physician, ensuring no significant health conditions were present.

### 2.3 Recruitment

A local media conference in August 2022 informed the Swiss public about the study. Interested individuals had an opportunity to register online, after which they were contacted via phone for an eligibility screening. They were then invited to a face-to-face interview with a study physician, in which detailed information about the study was provided, and written informed consent was obtained following the evaluation of inclusion and exclusion criteria. Recruitment of 374 participants started in September 2022 and completed in December 2022. Fourteen initially included participants dropped out from the study due to lack of interest or incomplete responses to the online questionnaire.

### 2.4 Data collection

Data sets were collected in January 2023 from 360 participants to gain sociodemographic information. Cannabis use days in the last 30 days and quantity of cannabis used per average use day (g) as well as cannabis use motives were evaluated via the Comprehensive Short Questionnaire of Current Cannabis Use (29). To facilitate the specification of cannabis quantity, participants were provided pictures of different amounts of cannabis flower and hashish in relation to a coin.

Depressive symptoms were assessed via the Patient Health Questionnaire depression scale (PHQ-9) (30), anxiety via the Generalized Anxiety Disorder Screener (GAD-7) (31), and ADHD via the World Health Organization (WHO) adult ADHD self-report scale (ASRS V1.1) (32). Psychosis was assessed via an adapted version of the Early Recognition Inventory (ERIraos) Checklist (33). This includes six items of the ERIraos on increased psychosis risk and early psychosis, respectively, and two additional items: “impression that certain occurrences are intended only for me” and “diagnosis of psychosis or schizophrenia by a medical professional, psychologist, or another health care professional in the last six months.” This tool is adept at identifying even nuanced psychotic symptoms essential for evaluating early cannabis-related psychopathology. Further substance use data was collected using the Cannabis Use Disorders Identification Test-Revised (CUDIT-R) (34) as well as the three-item version of the Alcohol Use Disorders Identification Test (AUDIT-C) (35). The use of other legal and illegal psychotropic substances was assessed via an adapted version of the Alcohol, Smoking and Substance Involvement Screening Test (ASSIST) (36). Specifically, the frequency of use during the last 6 months (0 never; 1 once or twice; 2 monthly; 3 weekly; 4 daily or almost daily) was asked for a set of 7 drug types corresponding to those used in the ASSIST.

### 2.5 Statistics

To examine the association between psychopathological scores and cannabis use, multiple linear regressions were computed. The following psychopathological scores served as independent variables: depressive symptom load (PHQ-9), anxiety symptom load (GAD-7), ADHD symptom load (ASRS-V1.1) and psychosis symptom load (ERIRaos). Each score was the sum of the single items of the respective scale. Cannabis use days in the past 30 days (in days) and cannabis quantity consumed in the past 30 days (in gram) served as dependent variables. The latter was computed by multiplying the number of reported use days by the reported average quantity consumed per day. Pearson correlations were initially used to assess the relationships between each of the four independent variables with each of the two dependent variables. Subsequently, by means of linear regressions, these eight correlations were adjusted for age, gender, education level, the AUDIT-C, and the ASSIST. Gender and education level were dummy coded with the categories ‘male’ and ‘university degree’ as the reference, respectively. AUDIT-C and ASSIST each formed a regressor after creating individual summarized scores across the three and seven items, respectively. Hence, a total of eight multiple linear regressions were computed.

To meet the assumptions of linear regression, particularly the normal distribution of residuals, both dependent variables and the four psychopathological scores were logarithmically calculated for inferential statistics. Analyses were conducted with R software version 4.2.1 (37). *p*-values were 2-sided, and statistical significance was set at α = 0.05.

## 3 Results

Study participants identified largely as male (80.8%), with a mean age of 35.8 years. Most participants had a university degree, lived alone, and were fully employed. A complete description of the sociodemographic profile of the study population can be found in Table 1. On average, participants consumed cannabis on nearly 19 days during the last 30 days with a mean use quantity of 1.46 grams per average use day. Results for cannabis consumption patterns, as well as psychopathological variables and other substance use, are presented in Table 2. Detailed results regarding cannabis use motives can be found in (Supplementary Figure S1).

**Table 1:** Sociodemographic characteristics of the participants (*n* = 360).

Gender	Female	63 (17.5%)
	Male	291 (80.8%)
	Non-binary	6 (1.7%)
Age in years	Mean (range)	35.8 (18–76)
Nationality	Swiss	269 (74.7%)
	Other	91 (25.3%)
Level of education	Obligatory schooling	34 (9.4%)
	Basic vocational education	110 (30.6%)
	University qualification	55 (15.3%)
	Higher vocational education	27 (7.5%)
	University degree	131 (36.4%)
	Other	3 (0.8%)
Form of living	Single person household	136 (37.8%)
	Couple without child(ren)	87 (24.2%)
	Couple with child(ren)	42 (11.7%)
	Single parent household with child(ren)	17 (4.7%)
	Other households with more than one person	78 (21.7%)
Employment	Employed, full-time	163 (45.3%)
	Employed, part-time (< 90%)	70 (19.4%)
	Student, full-time employed (90–100%)	7 (1.9%)
	Student, part-time employed (< 90%)	34 (9.4%)
	Unemployed	68 (18.9%)
	Economically inactive	18 (5.0%)

**Table 2:** Psychopathological symptom load, CUDIT-R, AUDIT-C, and other substance use and cannabis use patterns.

Assessment tools	Mean (SD)	Range	Probable condition (%)
PHQ-9 (depression)	5.30 (3.63)	0–19	3.06
GAD-7 (anxiety)	3.81 (3.39)	0–18	1.94
ASRS V1.1 (ADHD)	1.64 (1.50)	0–6	14.17
ERIaos (psychosis)	0.51 (0.93)	0–7	0.56
CUDIT-R (CUD)	11.09 (4.45)	1–27	33.9
AUDIT-C	3.71 (2.53)	0–12	
ASSIST adapted (Other Substance Use)	1.29 (2.10)	0–15	
Average cannabis use	30 days use quantity mean (SD)		18.73 (10.91)
	Average daily use quantity** mean (SD)		1.46 (2.84)
	Estimated use per month mean (SD)		34.27 (78.80)

Conditions indicating a high risk are based on the following cut-offs: ≥ 15 for potential severe depression (PHQ-9); ≥ 15 for potential severe anxiety disorder (GAD-7); ≥ 4 for potential ADHD (ASRS V1.1); ≥ 13 for potential problematic cannabis use (CUDIT-R). For psychosis the presented percentage represents the participants who were told to be schizophrenic or suffer from psychosis by a medical professional during the last 6 months. ADHD, Attention Deficit Hyperactivity Disorder; ASSIST, Alcohol, Smoking and Substance Involvement Screening Test; AUDIT-C, Alcohol Use Disorders Identification Test; CUD, Cannabis use disorder; CUDIT-R: Cannabis Use Disorder Identification Test – Revised; GAD-7, Generalized Anxiety Disorder Screener; PHQ-9, Patient Health Questionnaire depression scale; SD, Standard deviation. *During the last 30 days. **On an average use day.

### 3.1 Associations with psychopathological symptom load

Cannabis use frequency was significantly correlated with depression and anxiety scores (*r*(358) = 0.16, *p* = 0.003 and *r*(358) = 0.11, *p* = 0.034, respectively)). Correlations between depression, ADHD, as well as psychosis, and cannabis quantity were also statistically significant (*r*(358) = 0.15, *p* = 0.005; *r*(358) = 0.14, *p* = 0.008; *r*(358) = 0.16, *p* = 0.002, respectively). A trend for ADHD and psychosis was observed with cannabis use frequency (*r*(358) = 0.10, *p* = 0.060 and (*r*(358) = 0.09, *p* = 0.086). Anxiety also correlated marginally significant with cannabis use quantity (*r*(358) = 0.09, *p* = 0.098). The following significant relationships persisted in regression models after adjustment for the mentioned covariates.

The depression composite score was a significant predictor of cannabis use, specifically affecting the frequency (*p* = 0.006; β = 0.269; *t*(348) = 2.79; 95% CI [0.080, 0.458]), and quantity (*p* = 0.037; *β* = 0.350; *t*(348) = 2.09; 95% CI [0.022, 0.677]). Similarly, the ADHD scores significantly predicted cannabis use frequency (*p* = 0.034; *β* = 0.314; *t*(348) = 2.13; 95% CI [0.025, 0.603]); and quantity (*p* = 0.019; *β* = 0.599; *t*(348) = 2.36; 95% CI [0.102, 1.097]). The association between anxiety symptom load and cannabis frequency reached only marginal significance (*β* = 0.177; *t*(348) = 1.82; 95% CI [−0.014, 0.368]; *p* = 0.070). There were no significant associations between anxiety symptom load and cannabis quantity, nor for psychosis symptom load with any of the two consumption measures.

All education levels below university degree were significantly associated with higher frequency and quantity of cannabis use in all four psychopathological scores. A positive association was observed between quantity of cannabis use and use of other drugs, but not alcohol use. Detailed results from the regression models are presented below in Tables 3–6, which illustrate these associations and provide a more comprehensive view of the data.

**Table 3:** Relationship between depression and cannabis use frequency and quantity.

	Cannabis use frequency				Cannabis quantity			
	Estimate	SE	95% CI	*p*	Estimate	SE	95% CI	*p*
Depression								
Intercept	1.995	0.243	1.519–2.471	<0.001	1.031	0.420	0.207–1.854	0.015
PHQ-9 Score	0.269	0.097	0.080–0.458	0.006	0.350	0.167	0.022–0.677	0.037
Age	0.004	0.003	−0.001 – 0.010	0.107	0.006	0.005	−0.004 – 0.015	0.226
Gender (Reference.: Male)								
Female	−0.165	0.083	−0.327 – −0.004	0.046	−0.127	0.143	−0.407 – 0.153	0.374
Non-binary	0.344	0.234	−0.114 – 0.801	0.142	−0.490	0.404	−1.283 – 0.302	0.226
Education level (Reference: University Degree)								
Higher Vocational Education	0.284	0.120	0.049–0.519	0.018	0.492	0.208	0.086–0.899	0.018
University Qualification	0.367	0.092	0.186–0.548	<0.001	0.558	0.160	0.245–0.871	<0.001
Basic Vocational Education	0.428	0.073	0.285–0.571	<0.001	1.028	0.126	0.780–1.276	<0.001
Obligatory Schooling	0.388	0.111	0.171–0.605	<0.001	1.329	0.192	0.953–1.705	<0.001
Other	0.656	0.334	0.001–1.310	0.050	1.257	0.578	0.124–2.389	0.030
Additional substance use								
Alcohol Use	−0.017	0.012	−0.040 – 0.007	0.171	−0.037	0.021	−0.079 – 0.004	0.075
Other Drug Use	0.011	0.015	−0.018 – 0.039	0.470	0.059	0.025	0.010–0.109	0.020
	*R*-squared	0.152	*F*(11, 348)	5.690	*R*-squared	0.250	*F*(11, 348)	10.560
	adj. *R*-squared	0.126	*p*	<0.001	adj. *R*-squared	0.227	*p*	<0.001

CI, confidence interval; SE, standard error.

**Table 4:** Relationship between anxiety and cannabis use frequency and quantity.

	Cannabis use frequency				Cannabis quantity			
	Estimate	SE	95% CI	*p*	Estimate	SE	95% CI	*p*
Anxiety								
Intercept	2.182	0.239	1.714–2.651	<0.001	1.640	0.414	0.830–2.451	<0.001
GAD-7 Score	0.177	0.097	−0.014 – 0.368	0.070	0.027	0.169	−0.304 – 0.357	0.875
Age	0.005	0.003	−0.001 – 0.010	0.080	0.006	0.005	−0.003 – 0.015	0.220
Gender (Reference: Male)								
Female	−0.133	0.081	−0.292 – 0.027	0.103	−0.097	0.140	−0.371 – 0.177	0.488
Non-binary	0.396	0.233	−0.061 – 0.853	0.091	−0.432	0.401	−1.219 – 0.354	0.282
Education level (Reference: University Degree)								
Higher Vocational Education	0.304	0.122	0.066–0.543	0.013	0.487	0.210	0.075–0.900	0.021
University Qualification	0.373	0.093	0.191–0.554	<0.001	0.568	0.161	0.253–0.883	<0.001
Basic Vocational Education	0.429	0.074	0.285–0.573	<0.001	1.032	0.127	0.783–1.281	<0.001
Obligatory Schooling	0.394	0.112	0.174–0.614	<0.001	1.378	0.194	0.998–1.758	<0.001
Other	0.619	0.336	−0.039 – 1.276	0.066	1.139	0.581	0.000–2.277	0.051
Additional substance use								
Alcohol Use	−0.014	0.012	−0.037 – 0.010	0.265	−0.033	0.021	−0.074 – 0.008	0.116
Other Drug Use	0.013	0.015	−0.017 – 0.042	0.401	0.070	0.026	0.020–0.121	0.007
	*R*-squared	0.142	*F*(11, 348)	5.220	*R*-squared	0.241	*F*(11, 348)	10.040
	adj. *R*-squared	0.114	*p*	<0.001	adj. *R*-squared	0.217	*p*	<0.001

CI, confidence interval; SE, standard error.

**Table 5:** Relationship between ADHD and cannabis use frequency and quantity.

	Cannabis use frequency				Cannabis quantity			
	Estimate	SE	95% CI	*p*	Estimate	SE	95% CI	*p*
ADHD								
Intercept	1.961	0.301	1.370–2.551	<0.001	0.657	0.518	−0.359 – 1.672	0.206
ASRS-V.1.1 Score	0.314	0.148	0.025–0.603	0.034	0.599	0.254	0.102–1.097	0.019
Age	0.005	0.003	0.000–0.011	0.053	0.007	0.005	−0.002 – 0.017	0.115
Gender (Reference: Male)								
Female	−0.133	0.081	−0.292 – 0.027	0.103	−0.097	0.140	−0.371 – 0.177	0.488
Non-binary	0.396	0.233	−0.061 – 0.853	0.091	−0.432	0.401	−1.219 – 0.354	0.282
Education level (Reference: University Degree)								
Higher Vocational Education	0.288	0.121	0.052–0.524	0.017	0.505	0.207	0.098–0.911	0.015
University Qualification	0.382	0.093	0.200–0.564	<0.001	0.582	0.159	0.269–0.895	<0.001
Basic Vocational Education	0.441	0.074	0.297–0.585	<0.001	1.050	0.126	0.802–1.298	<0.001
Obligatory Schooling	0.400	0.111	0.183–0.618	<0.001	1.328	0.191	0.953–1.702	<0.001
Other	0.539	0.334	−0.115 – 1.192	0.107	1.093	0.574	−0.031 – 2.218	0.058
Additional substance use								
Alcohol Use	−0.016	0.012	−0.039 – 0.008	0.204	−0.037	0.021	−0.078 – 0.004	0.075
Other Drug Use	0.011	0.015	−0.018 – 0.041	0.440	0.055	0.026	0.005–0.105	0.031
	*R*-squared	0.145	*F*(11, 348)	5.350	*R*-squared	0.253	*F*(11, 348)	10.700
	adj. *R*-squared	0.118	*p*	<0.001	adj. *R*-squared	0.229	*p*	<0.001

CI, confidence interval; SE, standard error.

**Table 6:** Associations between psychosis and cannabis use frequency and quantity.

	Cannabis use frequency				Cannabis quantity			
	Estimate	SE	95% CI	*p*	Estimate	SE	95% CI	*p*
Psychosis								
Intercept	2.385	0.388	1.625–3.145	<0.001	1.034	0.667	−0.273 – 2.341	0.122
ERIraos (adapted) Score	0.072	0.219	−0.358 – 0.502	0.742	0.407	0.377	−0.331 – 1.146	0.281
Age	0.004	0.003	−0.001 – 0.010	0.103	0.006	0.005	−0.003 – 0.015	0.205
Gender (Reference: Male)								
Female	−0.115	0.081	−0.274 – 0.045	0.160	−0.068	0.140	−0.343 – 0.206	0.626
Non-binary	0.407	0.235	−0.055 – 0.868	0.085	−0.434	0.405	−1.228 – 0.359	0.284
Education level (Reference: University Degree)								
Higher Vocational Education	0.275	0.121	0.037–0.513	0.024	0.473	0.209	0.064–0.882	0.024
University Qualification	0.371	0.094	0.187–0.555	<0.001	0.547	0.162	0.230–0.864	<0.001
Basic Vocational Education	0.428	0.075	0.282–0.574	<0.001	1.013	0.128	0.761–1.264	<0.001
Obligatory Schooling	0.419	0.115	0.194–0.645	<0.001	1.328	0.198	0.941–1.715	<0.001
Other	0.562	0.336	−0.096 – 1.220	0.095	1.153	0.578	0.021–2.285	0.047
Additional substance use								
Alcohol Use	−0.013	0.012	−0.037 – 0.011	0.274	−0.034	0.021	−0.075 – 0.008	0.111
Other Drug Use	0.019	0.015	−0.009 – 0.048	0.185	0.068	0.025	0.019–0.117	0.007
	*R*-squared	0.134	*F*(11, 348)	4.880	*R*-squared	0.243	*F*(11, 348)	10.180
	adj. *R*-squared	0.106	*p*	<0.001	adj. *R*-squared	0.219	*p*	<0.001

CI, confidence interval; SE, standard error.

## 4 Discussion

### 4.1 Main findings

Our cross-sectional study elucidates the intricate relationship between psychopathological symptoms and cannabis use in nonclinical settings. After adjusting for important covariates, we observed significant positive associations between depressive symptoms and both cannabis use frequency and quantity. Similarly, ADHD symptoms were positively associated with cannabis use frequency and quantity. In contrast, associations with use frequency for anxiety and psychosis symptoms did not reach the significance level. The specific associations with different psychopathological variables highlight the need for a nuanced consideration of the factors influencing cannabis use. It is noteworthy that the likelihood of other mental illnesses was relatively low (with a probability of conditions ranging from <1 to 14%), in contrast to a 33.8% probability for the CUD, which was previously associated with increased mental health disorders among cannabis users (38).

The most commonly reported motivation for cannabis use among our participants was the pursuit of feeling good, providing additional context for these findings.

### 4.2 Psychopathologic symptom load and cannabis use

Our results are consistent with previous studies (26, 39, 40), that also reported a positive correlation between depression and cannabis use.

The associations between ADHD and cannabis use frequency were shown by previous studies, although none of them evaluated the use days on an interval scale (23, 41–43). Moreover, contrary to Fergusson et al., our outcomes remained significant after adjustment for other substance use as well (42). This suggests the possibility of an independent risk associated with cannabis consumption for ADHD symptom load among regular users, although the links between the two entities are complex and are beyond the scope of this paper (12, 44, 45). For example, a causal and temporal relationship for lifetime cannabis use was found in individuals with ADHD based on large data from genome-wide association studies with a two-sample Mendelian randomization approach. Individuals with ADHD were eight times likelier to use cannabis in their lifetime compared to those without the disorder (*p* = 0.00006) (44). A dose-dependent relationship was found for both hyperactive–impulsive and inattentive symptoms of ADHD with cannabis consumption (45). The co-occurrence of ADHD and CUD was discussed thoroughly in the literature (12).

While our findings align with much of the existing literature, there were some notable exceptions, particularly concerning the relationship between cannabis use and psychosis. Despite a large body of evidence connecting cannabis use to psychosis (6, 24, 46) our findings do not confirm this relationship, likely because of the exclusion of individuals suffering from acute psychosis. Similarly, the relationship between psychosis and high-potency cannabis was also not confirmed in a recent cross-sectional study with a similar study population to ours (4).

We found only marginal associations between anxiety and cannabis use. In a recent systematic review and meta-analysis of 10 studies, Xue et al. showed that cannabis use was associated with increased odds for the development of anxiety conditions (OR = 1.25; 95% CI, 1.01 to 1.54); however, no significant associations were found for GAD, social anxiety disorder, or panic disorder (8). Feingold et al. also did not find links between anxiety and cannabis use (47).

### 4.3 Cannabis use patterns

Cannabis consumption patterns observed in our study sample were similar to those reported by Caulkins et al. with an average monthly use of 34.27 g and a mean of 1.46 g use per day (16).Additionally, the mean days with cannabis use in the last 30 days of our sample (18.73) falls within the range of 15 to 23 days reported by the U.S. National Survey on Drug Use and Health (48, 49).

One third of the participants were classified at high risk for developing CUD, a rate that mirrors the one in three chance of cannabis dependence with weekly or more frequent usage as indicated by Leung et al. (17). However, it is important to note that while the systematic review and meta-analysis by Leung et al. reported a lower general estimate of 22% for developing CUD, they also documented a 13% prevalence of ‘cannabis addiction’ and a further 13% for ‘cannabis dependence’ (17). This comparative context underscores that our cohort’s risk level, despite a possibly higher than weekly consumption, is consistent with established findings for probable CUD occurrence.

The interactions between cannabis use frequency and quantity and CUD were highlighted in a recent analysis of over 3,000 participants (16). To reduce the likelihood of misjudgment of self-reported cannabis quantity by users (50), our study provided pictures of dosage examples in combination with quantity information in the questionnaire for the objective self-assessment (29). These methods were previously found to improve the accuracy of judgment (51).

Previous studies reported a negative impact of cannabis use on educational attainment at all levels (19, 52, 53). We further found that compared to a university degree, people with lower levels of education consume significantly more cannabis (both in terms of frequency and quantity). Other studies also showed that early-life cannabis use was associated with poor academic achievements and decline in Intelligence Quotient (IQ) (53–55). It is noteworthy to indicate that our sample had a higher level of education compared to the general population in Switzerland (56), which could have influenced the results to some degree.

Individuals with mental problems are known to consume cannabis more often, presumably for reasons of self-medication (57–60). However, the most common reason for cannabis use among our participants was “feeling good,” and only a minority reported help with concentration or psychological distress. This finding can again be explained by the fact that individuals with current severe mental disorders were excluded from the study.

The data imply that subclinical presentations of depression and ADHD could be driving higher consumption frequencies and volumes. This necessitates longitudinal studies to clarify whether consumption is a coping mechanism or a contributing factor to maintaining subclinical states.

Lastly, our findings indicated a positive association between cannabis quantity with other substance use. Similar links were shown between the frequency of cannabis consumption and the use of other substances (61). This pattern was notably evident in the consumption of amphetamines among participants with ADHD, which may indicate prescribed use. Such complexities reinforce the need for nuanced interpretations. These outcomes contribute to the already large body of evidence regarding frequent co-use of several illicit substances (62–64).

### 4.4 Study limitations, strengths and future directions

The cross-sectional design of our study necessitates a prudent approach to interpreting the findings precluding assessments of causality. Our sample mainly included psychologically healthy individuals motivated to participate in the pilot study, limiting the generalizability of our results and potentially contributing to discrepancies with other studies. The self-selection of participants and exclusion of individuals with acute psychosis or severe cognitive impairments further limited the sample’s representativeness.

However, these limitations are counterbalanced by several methodological strengths. First, we employed objective measures for quantifying cannabis use, including picture-based guidance for participant self-reporting. Second, our sample was drawn from a representative population of regular adult cannabis users in a major European city, which was validated by THC drug tests. Third, our analyses accounted for key confounding variables which were assessed by validated psychometric instruments, bolstering the potential independent effects of cannabis use on depressive and ADHD symptomatology. For the purposes of informing policy, the focus on a more representative population of regular cannabis users, as opposed to individuals with significant psychiatric comorbidities—who are generally advised against cannabis use—adds value to our findings.

Considering the findings of our study, future research should focus on longitudinal studies to explore causal relationships between cannabis use and psychopathological symptoms. A detailed examination of various aspects of cannabis consumption, including frequency, duration, and dosage, will provide deeper insights into its relationship with mental health disorders. Additionally, future studies should consider subgroups underrepresented in our research, such as individuals with existing mental health conditions, and investigate the role of socioeconomic and cultural factors in shaping cannabis use and its associated psychopathological symptoms. This approach will significantly deepen our understanding of the complex interplay between cannabis use and mental health.

## 5 Conclusion

In summary, we present a nuanced portrait of a predominantly psychologically healthy, drug-test-confirmed cohort of regular cannabis users. We observed a socially integrated, high-achieving sample with a low prevalence of CUD. The prevalence of other mental health disorders within this cohort was surprisingly low. Moreover, an association between depression and ADHD symptomatology with quantitative parameters of cannabis use was established, with no significant associations for anxiety and psychosis. These findings imply that subclinical presentations of depression and ADHD might be influencing higher consumption patterns, which underscores the importance of longitudinal studies to discern whether consumption functions as a coping mechanism or contributes to the maintenance of subclinical states. Our study advances the discussion on cannabis regulation, emphasizing the necessity of a nuanced approach in both public discourse and policy design, considering the complex relationship between cannabis use and mental health.

As the legal landscape surrounding cannabis continues to evolve, our findings emphasize the importance of focusing on evidence-based prevention, harm reduction, and safer use. Furthermore, this study’s findings contribute significantly to the ongoing discussions on cannabis policy and health regulation. By providing empirical data on the health impacts of regulated versus unregulated cannabis, this study supports the need for continued research and could inform future policy decisions aimed at improving public health outcomes in the context of cannabis legalization.

## Data availability statement

The original contributions presented in the study are included in the article/Supplementary material, further inquiries can be directed to the corresponding author.

## Ethics statement

The studies involving humans were approved by Ethikkommission Nordwest- und Zentralschweiz (EKNZ). The studies were conducted in accordance with the local legislation and institutional requirements. The participants provided their written informed consent to participate in this study.

## Author contributions

CFM: Investigation, Visualization, Writing – original draft, Writing – review & editing. LB-F: Writing – original draft, Writing – review & editing, Conceptualization, Data curation, Formal analysis, Methodology. JK: Data curation, Formal analysis, Methodology, Writing – original draft, Writing – review & editing. MM: Investigation, Writing – original draft, Writing – review & editing. AG: Investigation, Writing – review & editing. OH: Investigation, Writing – original draft. MV: Project administration, Resources, Writing – review & editing. MW: Conceptualization, Funding acquisition, Project administration, Supervision, Writing – original draft, Writing – review & editing. E-MP: Project administration, Supervision, Visualization, Writing – original draft, Writing – review & editing.
