# Supplementary figures and images for: Cannabis use and its association with psychopathological symptoms in a Swiss adult population: a cross-sectional analysis
Source: Front Public Health. 2024 May 22;12:1356988. doi: 10.3389/fpubh.2024.1356988 (PMC11151851; doi:10.3389/fpubh.2024.1356988)

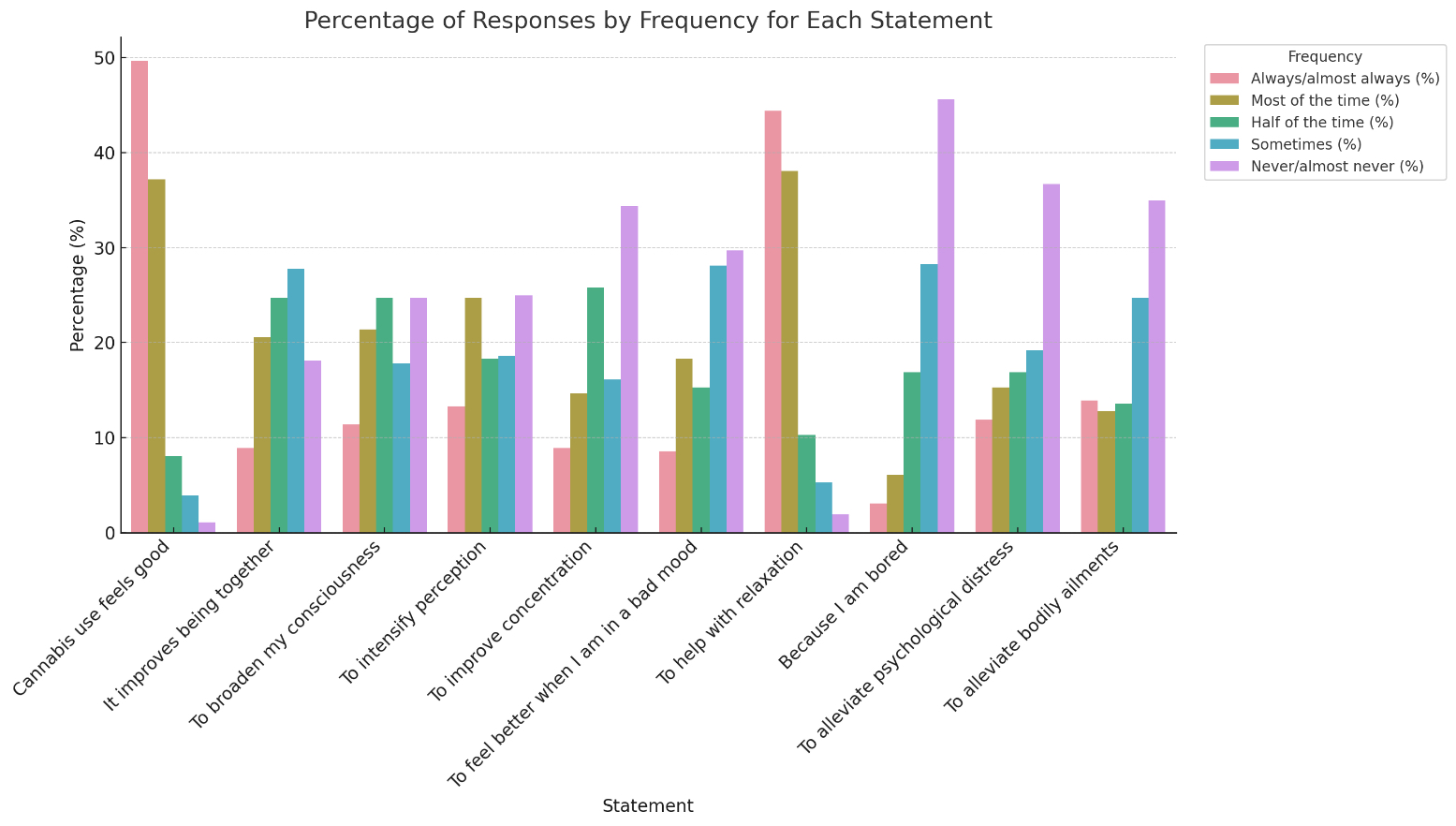

Supplement: Supplementary file 1 [file Image_1.JPEG]
